# Ethyl 6-methyl-3-(2-methyl­prop-1-en­yl)-2-oxo-4-phenyl-1,2,3,4-tetra­hydro­pyrimidine-5-carboxyl­ate

**DOI:** 10.1107/S1600536811042243

**Published:** 2011-10-22

**Authors:** Xi-Cun Wang, Xue-Hong Tang, Yu-Xia Da, Zhang Zhang, Zheng-Jun Quan

**Affiliations:** a Gansu Key Laboratory of Polymer Materials, College of Chemistry and Chemical Engineering, Northwest Normal University, Lanzhou 730070, People’s Republic of China

## Abstract

In the mol­ecule of the title compound, C_18_H_22_N_2_O_3_, the dihydro­pyrimidinone ring adopts an envelope conformation. The dihedral angle between the phenyl ring and the mean plane through the enamine fragment is 86.04 (7)°. The mol­ecular conformation is stabilized by an intra­molecular C—H⋯O hydrogen bond. In the crystal, inter­molecular N—H⋯O hydrogen bonds link pairs of mol­ecules into centrosymmetric dimers.

## Related literature

For general background to and pharmaceutical applications of pyrimidino­nes, see: Atwal (1990 ▶); Matsuda & Hirao (1965 ▶); Müller *et al.* (2008 ▶). For a related structure, see: Fun *et al.* (2009 ▶). For bond-length data, see: Allen *et al.* (1987 ▶).
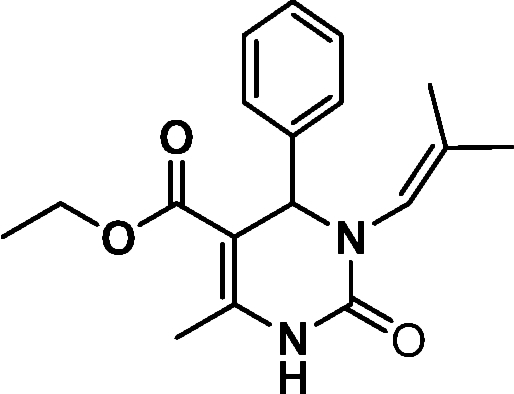


## Experimental

### 

#### Crystal data

C_18_H_22_N_2_O_3_
*M* *_r_* = 314.38
Monoclinic, 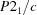

*a* = 14.114 (4) Å
*b* = 8.298 (2) Å
*c* = 14.629 (4) Å
β = 93.959 (2)°
*V* = 1709.3 (8) Å^3^
*Z* = 4
Mo *K*α radiation
μ = 0.08 mm^−1^
*T* = 296 K
0.25 × 0.24 × 0.22 mm

#### Data collection

Bruker APEXII CCD diffractometer
Absorption correction: multi-scan (*SADABS*; Bruker, 2008 ▶) *T*_min_ = 0.979, *T*_max_ = 0.982
11929 measured reflections
3180 independent reflections
2474 reflections with *I* > 2σ(*I*)
*R*_int_ = 0.020

#### Refinement

*R*[*F* ^2^ > 2σ(*F* ^2^)] = 0.050
*wR*(*F* ^2^) = 0.129
*S* = 0.96
3180 reflections
216 parameters
H atoms treated by a mixture of independent and constrained refinement
Δρ_max_ = 0.42 e Å^−3^
Δρ_min_ = −0.33 e Å^−3^

### 

Data collection: *APEX2* (Bruker, 2008 ▶); cell refinement: *SAINT* (Bruker, 2008 ▶); data reduction: *SAINT*; program(s) used to solve structure: *SHELXS97* (Sheldrick, 2008 ▶); program(s) used to refine structure: *SHELXL97* (Sheldrick, 2008 ▶); molecular graphics: *SHELXTL* (Sheldrick, 2008 ▶); software used to prepare material for publication: *SHELXTL*.

## Supplementary Material

Crystal structure: contains datablock(s) I, global. DOI: 10.1107/S1600536811042243/rz2648sup1.cif

Structure factors: contains datablock(s) I. DOI: 10.1107/S1600536811042243/rz2648Isup2.hkl

Supplementary material file. DOI: 10.1107/S1600536811042243/rz2648Isup3.cml

Additional supplementary materials:  crystallographic information; 3D view; checkCIF report

## Figures and Tables

**Table 1:** Hydrogen-bond geometry (Å, °)

*D*—H⋯*A*	*D*—H	H⋯*A*	*D*⋯*A*	*D*—H⋯*A*
C18—H18⋯O2	0.93	2.58	3.176 (3)	123
N1—H1⋯O1^i^	0.85 (2)	2.06 (2)	2.915 (2)	177 (2)

Symmetry code: (i) 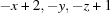.

**Fig. 1.**
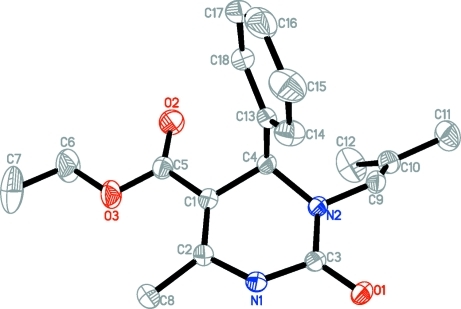
The molecular structure of the title compound with displacement ellipsoids drawn at the 30% probability level. H atoms are omitted.

**Fig. 2.**
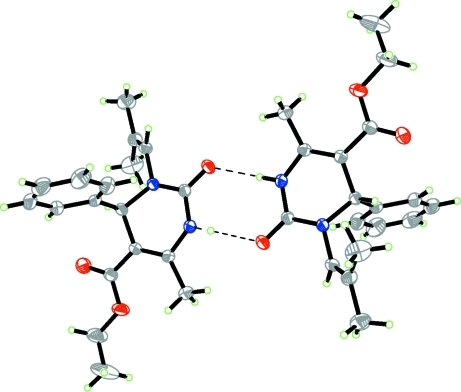
A view of a centrosymmetric dimeric unit formed via intermolecular hydrogen bonds (dashed lines) in the title compound. Displacement ellipsoids are drawn at the 30% probability level.

## supplementary crystallographic information

### Comment

3,4-Dihydropyrimidinones are compounds that have been drawn widespread attention due to their pharmaceutical applications. A variety of dihydropyrimidinone derivatives have been screened for antihypertension (Atwal, 1990) and antibacterial (Matsuda & Hirao, 1965) activities. At the same time, nitrogen-containing compounds, such as amines, enamines, and imines, are valuable and commercially important bulk chemicals, specialty chemicals, and pharmaceuticals (Müller *et al.*, 2008). As a result, dihydropyrimidin-2-ones-containing enamines can be synthesized by a new approach. As a continuation of our study on series of dihydropyrimidinone derivatives, we report herein the crystal structure of the title compound.

In the title compound (Fig. 1) bond lengths (Allen *et al.*, 1987) and angles are within normal ranges, and are comparable with those observed in a closely related structure (Fun *et al.*, 2009). The six-membered dihydropyrimidinone ring assumes an envelope conformation, with atom C4 displaced by 0.478 (2) Å from the mean plane of the other atoms. The dihedral angles formed by the mean plane through the enamine fragment (N2/C9–C12) and the phenyl ring is 86.04 (7)°. An intramolecular C—H···O hydrogen bond stabilizes the molecular conformation (Table 1). In the crystal, pairs of centrosymmetrically related molecules are linked by N—H···O hydrogen bonds into dimers (Fig. 2) generating rings of R_2_^2^(8) graph-set motif.

### Experimental

The title compound was synthesized by refluxing a mixture of 3,4-dihydropyrimidinone (1.0 mmol), isobutyraldehyde (2.0 mmol), and trimethylsilyl chloride (2.5 mmol) in anhydrous CH_2_Cl_2_ (10 ml) for 12 h. After completion of the reaction monitored by thin layer chromatography (TLC), the crude product was purified by column chromatography over silica gel with ethyl acetate/petroleum ether (1:1 *v*/*v* to afford the pure the title compound as the unique product. Crystals suitable for X-ray diffraction analysis were obtained on slow evaporation of an ethanol solution (yield 75%).

### Refinement

The H atom bound to the N atom of the dihydropyrimidinone ring was located in a difference Fourier map and refined freely. All other hydrogen atoms were placed in calculated positions with C—H = 0.93–0.98Å and included in the refinement in a riding-model approximation with *U*_iso_(H) = 1.2 *U*_eq_(C) or 1.5 *U*_eq_(C) for methyl H atoms.

### Crystal data

**Table:**

C_18_H_22_N_2_O_3_	*F*(000) = 672
*M**_r_* = 314.38	*D*_x_ = 1.222 Mg m^−^^3^
Monoclinic, *P*2_1_/*c*	Mo *K*α radiation, λ = 0.71073 Å
Hall symbol: -P 2ybc	Cell parameters from 4330 reflections
*a* = 14.114 (4) Å	θ = 2.8–26.4°
*b* = 8.298 (2) Å	µ = 0.08 mm^−^^1^
*c* = 14.629 (4) Å	*T* = 296 K
β = 93.959 (2)°	Block, colourless
*V* = 1709.3 (8) Å^3^	0.25 × 0.24 × 0.22 mm
*Z* = 4	

### Data collection

**Table:**

Bruker APEXII CCD diffractometer	3180 independent reflections
Radiation source: fine-focus sealed tube	2474 reflections with *I* > 2σ(*I*)
graphite	*R*_int_ = 0.020
φ and ω scans	θ_max_ = 25.5°, θ_min_ = 2.8°
Absorption correction: multi-scan (*SADABS*; Bruker, 2008)	*h* = −16→17
*T*_min_ = 0.979, *T*_max_ = 0.982	*k* = −10→8
11929 measured reflections	*l* = −17→17

### Refinement

**Table:**

Refinement on *F*^2^	Primary atom site location: structure-invariant direct methods
Least-squares matrix: full	Secondary atom site location: difference Fourier map
*R*[*F*^2^ > 2σ(*F*^2^)] = 0.050	Hydrogen site location: inferred from neighbouring sites
*wR*(*F*^2^) = 0.129	H atoms treated by a mixture of independent and constrained refinement
*S* = 0.96	*w* = 1/[σ^2^(*F*_o_^2^) + (0.0463*P*)^2^ + 1.3266*P*] where *P* = (*F*_o_^2^ + 2*F*_c_^2^)/3
3180 reflections	(Δ/σ)_max_ = 0.001
216 parameters	Δρ_max_ = 0.42 e Å^−^^3^
0 restraints	Δρ_min_ = −0.33 e Å^−^^3^

### Special details

**Table:**

Geometry. All e.s.d.'s (except the e.s.d. in the dihedral angle between two l.s. planes) are estimated using the full covariance matrix. The cell e.s.d.'s are taken into account individually in the estimation of e.s.d.'s in distances, angles and torsion angles; correlations between e.s.d.'s in cell parameters are only used when they are defined by crystal symmetry. An approximate (isotropic) treatment of cell e.s.d.'s is used for estimating e.s.d.'s involving l.s. planes.
Refinement. Refinement of *F*^2^ against ALL reflections. The weighted *R*-factor *wR* and goodness of fit *S* are based on *F*^2^, conventional *R*-factors *R* are based on *F*, with *F* set to zero for negative *F*^2^. The threshold expression of *F*^2^ > σ(*F*^2^) is used only for calculating *R*-factors(gt) *etc*. and is not relevant to the choice of reflections for refinement. *R*-factors based on *F*^2^ are statistically about twice as large as those based on *F*, and *R*- factors based on ALL data will be even larger.

### Fractional atomic coordinates and isotropic or equivalent isotropic displacement parameters (Å^2^)

**Table:**

	*x*	*y*	*z*	*U*_iso_*/*U*_eq_	
C1	0.72325 (13)	0.1486 (2)	0.41220 (12)	0.0366 (4)	
C2	0.77888 (13)	0.0257 (2)	0.44302 (13)	0.0388 (4)	
C3	0.92081 (13)	0.1942 (2)	0.44340 (12)	0.0371 (4)	
C4	0.76336 (12)	0.3183 (2)	0.41569 (13)	0.0365 (4)	
H4	0.7332	0.3778	0.3636	0.044*	
C5	0.62397 (14)	0.1386 (3)	0.37604 (14)	0.0437 (5)	
C6	0.48677 (19)	−0.0234 (4)	0.3462 (2)	0.0869 (10)	
H6A	0.4503	0.0676	0.3662	0.104*	
H6B	0.4827	−0.0246	0.2798	0.104*	
C7	0.4492 (2)	−0.1689 (5)	0.3797 (2)	0.1148 (14)	
H7A	0.4801	−0.2590	0.3534	0.172*	
H7B	0.3822	−0.1738	0.3631	0.172*	
H7C	0.4598	−0.1723	0.4452	0.172*	
C8	0.75390 (16)	−0.1477 (3)	0.45539 (18)	0.0582 (6)	
H8A	0.6965	−0.1551	0.4867	0.087*	
H8B	0.8045	−0.2006	0.4909	0.087*	
H8C	0.7447	−0.1986	0.3965	0.087*	
C9	0.91126 (13)	0.4621 (2)	0.38297 (14)	0.0423 (5)	
H9	0.9533	0.5070	0.4277	0.051*	
C10	0.89706 (15)	0.5394 (3)	0.30492 (15)	0.0497 (5)	
C11	0.9448 (2)	0.7003 (3)	0.2919 (2)	0.0760 (8)	
H11A	0.9780	0.7327	0.3485	0.114*	
H11B	0.8977	0.7796	0.2738	0.114*	
H11C	0.9891	0.6907	0.2453	0.114*	
C12	0.8374 (2)	0.4801 (4)	0.22492 (17)	0.0868 (9)	
H12A	0.8216	0.3690	0.2342	0.130*	
H12B	0.8717	0.4900	0.1707	0.130*	
H12C	0.7802	0.5427	0.2179	0.130*	
C13	0.73992 (13)	0.4064 (2)	0.50270 (14)	0.0406 (5)	
C14	0.79880 (18)	0.4024 (3)	0.58146 (16)	0.0579 (6)	
H14	0.8564	0.3481	0.5816	0.070*	
C15	0.7738 (2)	0.4779 (3)	0.66064 (19)	0.0778 (8)	
H15	0.8146	0.4739	0.7133	0.093*	
C16	0.6894 (2)	0.5584 (3)	0.6617 (2)	0.0795 (9)	
H16	0.6726	0.6086	0.7150	0.095*	
C17	0.6301 (2)	0.5647 (3)	0.5843 (2)	0.0730 (8)	
H17	0.5726	0.6193	0.5849	0.088*	
C18	0.65490 (15)	0.4902 (3)	0.50460 (18)	0.0551 (6)	
H18	0.6143	0.4964	0.4519	0.066*	
N1	0.87357 (11)	0.0582 (2)	0.46786 (12)	0.0415 (4)	
N2	0.86624 (10)	0.31325 (18)	0.40381 (11)	0.0374 (4)	
O1	1.00791 (9)	0.20302 (17)	0.45540 (10)	0.0488 (4)	
O2	0.57967 (10)	0.2533 (2)	0.34516 (12)	0.0628 (5)	
O3	0.58555 (10)	−0.0075 (2)	0.38094 (12)	0.0618 (5)	
H1	0.9093 (16)	−0.018 (3)	0.4888 (15)	0.052 (6)*	

### Atomic displacement parameters (Å^2^)

**Table:**

	*U*^11^	*U*^22^	*U*^33^	*U*^12^	*U*^13^	*U*^23^
C1	0.0337 (10)	0.0370 (10)	0.0396 (10)	−0.0014 (8)	0.0047 (8)	0.0009 (8)
C2	0.0366 (10)	0.0362 (10)	0.0439 (10)	−0.0029 (8)	0.0062 (8)	0.0002 (8)
C3	0.0342 (10)	0.0368 (10)	0.0408 (10)	0.0020 (8)	0.0050 (8)	0.0024 (8)
C4	0.0295 (9)	0.0357 (10)	0.0444 (10)	0.0011 (8)	0.0028 (7)	0.0067 (8)
C5	0.0379 (11)	0.0466 (12)	0.0467 (11)	−0.0047 (9)	0.0040 (8)	−0.0024 (9)
C6	0.0550 (16)	0.082 (2)	0.120 (2)	−0.0265 (15)	−0.0249 (16)	0.0113 (18)
C7	0.086 (2)	0.162 (4)	0.094 (2)	−0.071 (2)	−0.0088 (18)	0.022 (2)
C8	0.0497 (13)	0.0388 (12)	0.0861 (17)	−0.0040 (10)	0.0037 (12)	0.0065 (11)
C9	0.0349 (10)	0.0401 (11)	0.0520 (12)	−0.0039 (8)	0.0029 (8)	0.0080 (9)
C10	0.0520 (12)	0.0454 (12)	0.0524 (12)	0.0005 (10)	0.0096 (10)	0.0089 (10)
C11	0.0805 (18)	0.0615 (16)	0.0869 (19)	−0.0118 (14)	0.0118 (15)	0.0278 (14)
C12	0.125 (3)	0.087 (2)	0.0479 (14)	−0.0181 (19)	0.0008 (15)	0.0073 (14)
C13	0.0396 (10)	0.0284 (10)	0.0548 (12)	−0.0030 (8)	0.0101 (9)	0.0036 (8)
C14	0.0628 (14)	0.0543 (14)	0.0564 (13)	0.0086 (12)	0.0015 (11)	−0.0065 (11)
C15	0.106 (2)	0.0690 (18)	0.0584 (15)	0.0009 (17)	0.0081 (15)	−0.0119 (13)
C16	0.106 (2)	0.0566 (17)	0.081 (2)	−0.0116 (16)	0.0432 (18)	−0.0182 (14)
C17	0.0653 (16)	0.0461 (14)	0.112 (2)	0.0006 (12)	0.0415 (16)	−0.0130 (15)
C18	0.0432 (12)	0.0420 (12)	0.0813 (16)	0.0004 (10)	0.0141 (11)	−0.0041 (11)
N1	0.0340 (9)	0.0335 (9)	0.0569 (10)	0.0033 (7)	0.0017 (7)	0.0096 (8)
N2	0.0305 (8)	0.0344 (9)	0.0476 (9)	0.0014 (7)	0.0051 (6)	0.0078 (7)
O1	0.0296 (7)	0.0477 (9)	0.0691 (9)	0.0014 (6)	0.0025 (6)	0.0112 (7)
O2	0.0429 (8)	0.0586 (10)	0.0841 (11)	0.0027 (8)	−0.0149 (8)	0.0068 (9)
O3	0.0425 (8)	0.0567 (10)	0.0846 (12)	−0.0162 (7)	−0.0085 (8)	0.0058 (8)

### Geometric parameters (Å, °)

**Table:**

C1—C2	1.346 (3)	C9—C10	1.313 (3)
C1—C5	1.465 (3)	C9—N2	1.432 (2)
C1—C4	1.517 (3)	C9—H9	0.9300
C2—N1	1.387 (2)	C10—C12	1.478 (3)
C2—C8	1.495 (3)	C10—C11	1.514 (3)
C3—O1	1.232 (2)	C11—H11A	0.9600
C3—N2	1.357 (2)	C11—H11B	0.9600
C3—N1	1.371 (2)	C11—H11C	0.9600
C4—N2	1.475 (2)	C12—H12A	0.9600
C4—C13	1.524 (3)	C12—H12B	0.9600
C4—H4	0.9800	C12—H12C	0.9600
C5—O2	1.209 (2)	C13—C14	1.374 (3)
C5—O3	1.332 (3)	C13—C18	1.389 (3)
C6—C7	1.419 (4)	C14—C15	1.384 (3)
C6—O3	1.457 (3)	C14—H14	0.9300
C6—H6A	0.9700	C15—C16	1.366 (4)
C6—H6B	0.9700	C15—H15	0.9300
C7—H7A	0.9600	C16—C17	1.362 (4)
C7—H7B	0.9600	C16—H16	0.9300
C7—H7C	0.9600	C17—C18	1.386 (4)
C8—H8A	0.9600	C17—H17	0.9300
C8—H8B	0.9600	C18—H18	0.9300
C8—H8C	0.9600	N1—H1	0.85 (2)
			
C2—C1—C5	126.84 (18)	C9—C10—C11	119.8 (2)
C2—C1—C4	118.95 (16)	C12—C10—C11	115.4 (2)
C5—C1—C4	114.19 (16)	C10—C11—H11A	109.5
C1—C2—N1	118.06 (17)	C10—C11—H11B	109.5
C1—C2—C8	129.24 (18)	H11A—C11—H11B	109.5
N1—C2—C8	112.70 (17)	C10—C11—H11C	109.5
O1—C3—N2	123.30 (17)	H11A—C11—H11C	109.5
O1—C3—N1	120.66 (17)	H11B—C11—H11C	109.5
N2—C3—N1	116.02 (16)	C10—C12—H12A	109.5
N2—C4—C1	109.75 (15)	C10—C12—H12B	109.5
N2—C4—C13	112.60 (15)	H12A—C12—H12B	109.5
C1—C4—C13	111.80 (15)	C10—C12—H12C	109.5
N2—C4—H4	107.5	H12A—C12—H12C	109.5
C1—C4—H4	107.5	H12B—C12—H12C	109.5
C13—C4—H4	107.5	C14—C13—C18	118.0 (2)
O2—C5—O3	122.32 (18)	C14—C13—C4	122.37 (18)
O2—C5—C1	123.19 (19)	C18—C13—C4	119.65 (19)
O3—C5—C1	114.49 (18)	C13—C14—C15	121.1 (2)
C7—C6—O3	109.2 (3)	C13—C14—H14	119.4
C7—C6—H6A	109.8	C15—C14—H14	119.4
O3—C6—H6A	109.8	C16—C15—C14	120.2 (3)
C7—C6—H6B	109.8	C16—C15—H15	119.9
O3—C6—H6B	109.8	C14—C15—H15	119.9
H6A—C6—H6B	108.3	C17—C16—C15	119.7 (3)
C6—C7—H7A	109.5	C17—C16—H16	120.1
C6—C7—H7B	109.5	C15—C16—H16	120.1
H7A—C7—H7B	109.5	C16—C17—C18	120.4 (3)
C6—C7—H7C	109.5	C16—C17—H17	119.8
H7A—C7—H7C	109.5	C18—C17—H17	119.8
H7B—C7—H7C	109.5	C17—C18—C13	120.6 (2)
C2—C8—H8A	109.5	C17—C18—H18	119.7
C2—C8—H8B	109.5	C13—C18—H18	119.7
H8A—C8—H8B	109.5	C3—N1—C2	124.64 (17)
C2—C8—H8C	109.5	C3—N1—H1	114.6 (15)
H8A—C8—H8C	109.5	C2—N1—H1	119.2 (15)
H8B—C8—H8C	109.5	C3—N2—C9	118.14 (15)
C10—C9—N2	124.28 (19)	C3—N2—C4	120.31 (15)
C10—C9—H9	117.9	C9—N2—C4	117.11 (15)
N2—C9—H9	117.9	C5—O3—C6	116.56 (19)
C9—C10—C12	124.8 (2)		
			
C5—C1—C2—N1	174.98 (18)	C15—C16—C17—C18	−0.1 (4)
C4—C1—C2—N1	−6.5 (3)	C16—C17—C18—C13	0.8 (4)
C5—C1—C2—C8	−5.1 (4)	C14—C13—C18—C17	−1.1 (3)
C4—C1—C2—C8	173.4 (2)	C4—C13—C18—C17	176.9 (2)
C2—C1—C4—N2	31.5 (2)	O1—C3—N1—C2	−168.31 (18)
C5—C1—C4—N2	−149.80 (16)	N2—C3—N1—C2	10.0 (3)
C2—C1—C4—C13	−94.2 (2)	C1—C2—N1—C3	−16.6 (3)
C5—C1—C4—C13	84.5 (2)	C8—C2—N1—C3	163.42 (19)
C2—C1—C5—O2	−176.1 (2)	O1—C3—N2—C9	−6.1 (3)
C4—C1—C5—O2	5.3 (3)	N1—C3—N2—C9	175.65 (16)
C2—C1—C5—O3	4.7 (3)	O1—C3—N2—C4	−161.77 (18)
C4—C1—C5—O3	−173.87 (17)	N1—C3—N2—C4	20.0 (3)
N2—C9—C10—C12	−4.0 (4)	C10—C9—N2—C3	134.3 (2)
N2—C9—C10—C11	177.0 (2)	C10—C9—N2—C4	−69.3 (3)
N2—C4—C13—C14	−33.4 (3)	C1—C4—N2—C3	−38.9 (2)
C1—C4—C13—C14	90.7 (2)	C13—C4—N2—C3	86.3 (2)
N2—C4—C13—C18	148.68 (18)	C1—C4—N2—C9	165.15 (16)
C1—C4—C13—C18	−87.2 (2)	C13—C4—N2—C9	−69.6 (2)
C18—C13—C14—C15	0.7 (3)	O2—C5—O3—C6	0.5 (3)
C4—C13—C14—C15	−177.2 (2)	C1—C5—O3—C6	179.7 (2)
C13—C14—C15—C16	0.0 (4)	C7—C6—O3—C5	−163.3 (3)
C14—C15—C16—C17	−0.3 (4)		

### Hydrogen-bond geometry (Å, °)

**Table:**

*D*—H···*A*	*D*—H	H···*A*	*D*···*A*	*D*—H···*A*
C18—H18···O2	0.93	2.58	3.176 (3)	123.
N1—H1···O1^i^	0.85 (2)	2.06 (2)	2.915 (2)	177 (2)

Symmetry codes: (i) −*x*+2, −*y*, −*z*+1.
